# Antituberculous Drug-Induced Hepatitis in a Patient With Congenital Adrenal Hyperplasia: A Clinical Challenge

**DOI:** 10.7759/cureus.25557

**Published:** 2022-06-01

**Authors:** Mudassir Shafique, Ahsan Tameez-ud-din, Asim Tameez Ud Din, Farooq Mohyud Din Chaudhary, Awais A Bhatti

**Affiliations:** 1 Pulmonology, Islamabad Medical Complex, Islamabad, PAK; 2 Internal Medicine, Islamabad Medical Complex, Islamabad, PAK; 3 Internal Medicine, Sisters of Charity Hospital, Buffalo, USA; 4 Department of Gastroenterology, Royal Berkshire Hospital, Reading, GBR; 5 Internal Medicine, Rawalpindi Medical University, Rawalpindi, PAK

**Keywords:** drug toxicity, congenital adrenal hyperplasia, drug-induced hepatitis, antitubercular antibiotics, tuberculosis

## Abstract

Pulmonary tuberculosis (TB) is highly prevalent in Pakistan, and immunosuppressed individuals (including those on long-term corticosteroid therapy) are at an especially high risk of infection. Owing to the limited number of effective antituberculous drugs, treating resistant cases or patients who develop unfavorable side effects from the first-line agents becomes a daunting task. We discuss a patient with congenital adrenal hyperplasia (CAH) suffering from pulmonary TB who developed drug-induced hepatitis after being started on recommended first-line anti-TB drugs.

## Introduction

Congenital adrenal hyperplasia (CAH) is a rare group of disorders involving the deficiency of the key enzymes involved in the synthesis of certain endogenous steroids. More than 90% of the patients have a deficiency of the 21-hydroxylase enzyme, which results in salt wasting, growth retardation, and the inability of the body to cope with highly stressful conditions (such as disease and trauma) [1]. The management of this condition includes long-term mineralocorticoid and glucocorticoid replacement, which increases the risk of infection in these patients [2].

Pakistan has a very high disease burden of pulmonary tuberculosis (TB), and immunosuppressed individuals are at an especially high risk of getting infected by this disease. The Mantoux test is an inexpensive test used for the initial screening of patients with TB, but it has a low specificity in immunosuppressed or vaccinated individuals. There are more specific investigations such as the QuantiFERON-TB Gold test and sputum Gene Xpert, which have a high sensitivity and specificity and are now replacing the traditional skin prick tests [3].

In this case report, we discuss a patient with congenital adrenal hyperplasia who developed pulmonary TB (with a negative initial skin prick test).

## Case presentation

**Figure 1:**
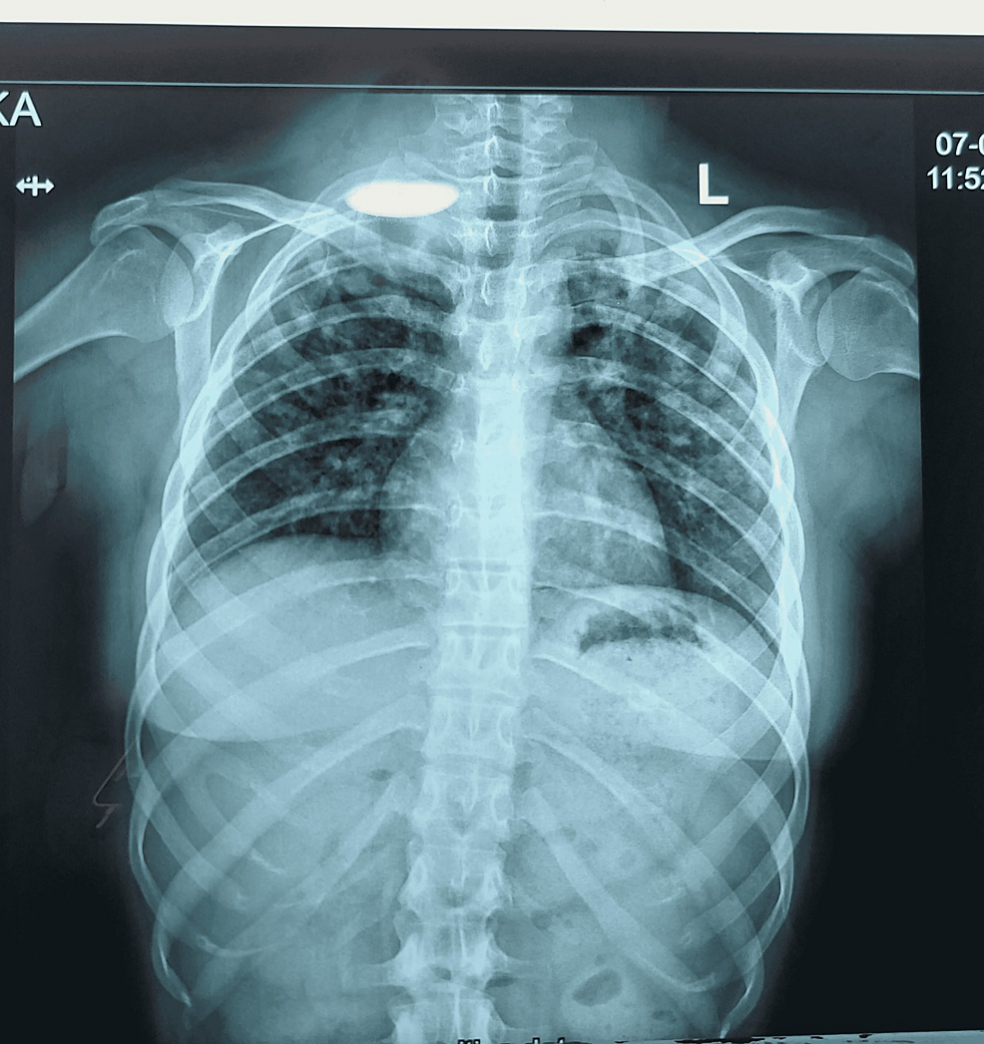
Chest X-ray showing diffuse bilateral infiltrates.

**Figure 2:**
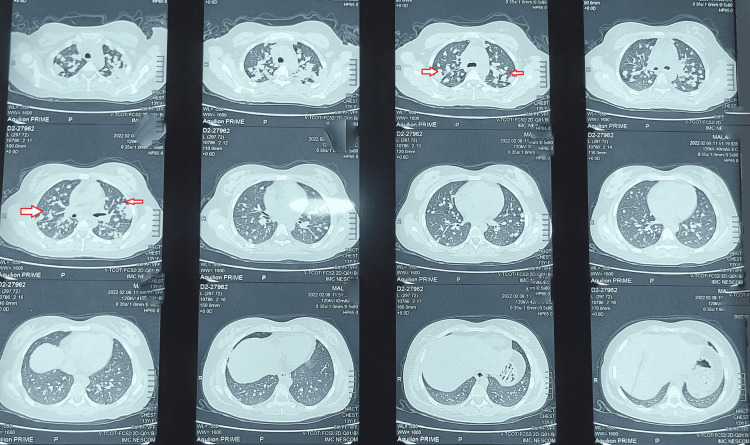
Chest CT scan showing infiltrates in both lung fields. Arrows point toward multiple diffuse infiltrates.

A 13-year-old female presented with a productive cough, low-grade fever, and night sweats for three weeks. She was diagnosed case of congenital adrenal hyperplasia (21-hydroxylase variant). The patient had had two surgeries for her ambiguous genitals (at age one year and six years) and was on long-term mineralocorticoid (fludrocortisone) and glucocorticoid (hydrocortisone) replacement therapy. Her baseline laboratory investigations (including liver function tests (LFTs)) were normal except for ESR, which was raised (75 mm/hour; normal range: 0-20 mm/hour). The patient’s chest X-ray showed bilateral diffuse infiltrates (Figure 1). Her Mantoux test was negative as was her sputum AFB test. A high-resolution CT scan of the chest was done, which corroborated the findings of the chest X-ray with diffuse bilateral opacities in both lung fields (Figure 2).

Considering her immunosuppressed state, symptoms, and socioeconomic status, pulmonary tuberculosis was the primary provisional diagnosis. COVID-19 infection was considered a differential diagnosis, but she tested negative for coronavirus disease, and her TB Gene Xpert was ordered, which was found to be positive. This test also revealed no rifampicin resistance.

After confirming the diagnosis of pulmonary TB clinically, radiologically, and hematologically, she was started on the classical antituberculous treatment (ATT) regimen involving a fixed drug combination of isoniazid (INH), rifampicin (RIF), pyrazinamide (PZA), and ethambutol (EMB) according to her weight (INH: 5 mg/kg, RIF: 10 mg/kg, PZA: 25 mg/kg, EMB: 15 mg/kg once daily) along with vitamin B6. Her baseline laboratory investigations were normal, and follow-up after one week showed that the patient was responding well to the therapy and that symptoms were improving.

The patient presented to the hospital after two weeks of therapy with sudden onset of epigastric discomfort and vomiting. Some of her liver function tests (LFTs) were found to be deranged (Table 1). Her hepatitis B and C antibody tests were negative, and no other cause of deranged liver function tests was found. A diagnosis of ATT-induced hepatitis was made, and all antituberculous medications were stopped. Her serial LFTs over the next week showed an improving trend, and she was gradually restarted on a modified ATT regimen, which excluded PZA. The patient was kept under observation for two weeks, at the end of which she had been restarted on INH, RIF, and EMB. Her LFTs stayed within the normal range during the course of the hospital stay, and she remained asymptomatic throughout this time. The patient was discharged with strict advice regarding drug dosage and how to contact the hospital immediately in case of recurrence of symptoms. Her routine follow-up after two weeks showed that the patient was responding well to the treatment. She was sent home after advice regarding strict adherence to the compliance of medication and follow-up laboratory investigations every month.

**Table 1:** Liver function tests of the patient with reference values.

Liver function test	Patient’s values	Reference range
Alanine aminotransferase (ALT)	340 U/L	5-50 U/L
Aspartate aminotransferase (AST)	234 U/L	5-34 U/L
Alkaline phosphatase (ALP)	76 U/L	<500 U/L in 1-12 years, <750 U/L in 12-15 years, and 40-150 U/L in >20 years
Total bilirubin	1.1 mg/dL	0.2-1.1 mg/dL

## Discussion

Pakistan ranks fifth in the overall disease burden of TB, with pulmonary TB accounting for a majority of cases [3,4]. Pulmonary TB usually presents with persistent low-grade fever, productive cough (may have blood in sputum), night sweats, weakness, and weight loss. The traditional skin prick tests are considered an inexpensive initial screening tool for TB and are still widely used in Pakistan. The interpretation of these tests becomes challenging in immunosuppressed individuals, and a false-negative test is a likely outcome in such cases [5]. This was the case with our patient, and the final diagnosis was made through more specific laboratory investigations.

TB has aptly been termed as the disease of poverty in literature as the major contributing factors to its spread are associated with poor socioeconomic status [6]. Owing to its high prevalence in this country, immunosuppressed and malnourished individuals are at a high risk of catching this disease, especially if they belong to the lower socioeconomic strata of society. Congenital adrenal hyperplasia is a condition where the body produces insufficient quantities of essential steroids. Patients with CAH have an increased risk of infections, and special care should be taken to avoid high-risk situations [2,7].

During the past decade, Pakistan has made appreciable efforts to tackle the menace of TB through a dedicated national disease control program, but the emergence of highly resistant strains and the limited number of drugs are some of the major roadblocks in the path toward achieving its goals [3]. The long duration of treatment gives rise to problems of noncompliance and non-affordability of the drugs, which adds to the challenges faced by health authorities. Although the directly observed treatment (DOT) introduced by the WHO and the simplification of the dosage of the treatment regimen has proved to improve compliance, there is still a long way to go before complete eradication of this disease can be achieved [4,8].

The most commonly used drug regimen for pulmonary TB includes four drugs (isoniazid, rifampicin, ethambutol, and pyrazinamide) initially for two months and then two drugs (isoniazid and rifampicin) for the next four months. This classic antituberculous regimen, although highly effective, can result in serious side effects including hepatotoxicity, vision defects, and neuropathy [9].

Drug-induced hepatitis is essentially a diagnosis of exclusion, and the main criteria for diagnosing this condition include a more than twofold increase in serum alanine aminotransferase (ALT) elevation within months of initiating a drug and a fall in the serum levels of this enzyme after the discontinuation of the offending medicine. Antituberculosis drugs have been associated with this condition, and the prevalence of drug-induced liver injury has been reported in up to a quarter of patients receiving an antituberculous treatment regimen [10]. The mechanism of hepatotoxicity varies from the generation of free radicals (INH and PZA) to the inhibition of the major bile salt exporter pump (RIF) [11]. Pyrazinamide is widely considered to be the most hepatotoxic drug among the classic anti-TB drug regimen, and its discontinuation may lead to the normalization of liver functions. The American Thoracic Society recommends restarting treatment in such patients without PZA with a regimen including INH, RIF, and EMB for nine months in susceptible individuals. The patients’ liver functions should be serially monitored for improvement or deterioration [11].

The emergence of variants that are resistant to the ubiquitously prescribed first-line drugs is a cause of further concern due to the limited number of effective anti-TB drugs available. Numerous international guidelines for the reintroduction of anti-TB drugs in case of discontinuation of the classic regimen owing to unfavorable side effects (ATT-induced hepatitis in this case) exist, but it is still a daunting task due to the limited number of alternatives and their unsuitable safety profile [10,11]. Scientists and health authorities have long called for the development of new, safer, and more effective drugs for TB, but the incentives for new developments in this field remain scarce, and there has been limited advancement in the development of efficacious and safe TB drugs [12]. The encouraging results from trials involving some new drugs (e.g., bedaquiline) have been received with cautious enthusiasm by the healthcare community, and only time will tell whether these results can be translated into a real-world benefit to the patients and the physicians trying to tackle this disease head-on [12,13].

## Conclusions

This report describes a clinically challenging case of diagnosing and treating pulmonary TB in an immunosuppressed individual. While this article briefly discusses the importance of relying on specific TB tests instead of the traditional screening tests (skin prick tests), the main emphasis is on the limited number of available TB drugs and the need to discover new, safer, and more effective antituberculosis drugs. The development of effective COVID-19 vaccinations within a miraculously brief span of time proves that if properly incentivized, the pharmaceutical companies can come up with better alternatives to the available drugs in a short span of time. The research involving TB and its treatment has largely remained stagnant over the past few decades, and international collaborative efforts are the need of the hour in order to rid the world of this menace before the health sectors of less developed countries like Pakistan capitulate to this disease of consumption.
